# Incidence of COVID-19 and Influenza-Related Outcomes and Vaccinations in the United States, October 2022 Through December 2024

**DOI:** 10.3390/vaccines14050424

**Published:** 2026-05-08

**Authors:** Heather R. Hensler, Tianyi Lu, Yoonyoung Park, Machaon Bonafede, Isabelle Winer, Christopher Adams, Keya Joshi, Amanda Wilson

**Affiliations:** 1 Moderna, Inc., Cambridge, MA 02142, USA; tianyi.lu@modernatx.com (T.L.); yoonyoung.park@modernatx.com (Y.P.); keya.joshi@modernatx.com (K.J.); amanda.wilson@modernatx.com (A.W.); 2 Veradigm, Chicago, IL 60654, USA; mac.bonafede@veradigm.com (M.B.); isabelle.winer@veradigm.com (I.W.); christopher.adams@veradigm.com (C.A.)

**Keywords:** COVID-19, influenza, vaccination, hospitalization, real-world evidence, mRNA vaccines

## Abstract

Background/Objectives: We still do not clearly know whether COVID-19 continues to impose a greater clinical burden than influenza in the “post-pandemic” era. Our study quantified and compared monthly COVID-19 and influenza hospitalization incidence among adult subgroups from October 2022 through December 2024. We assessed vaccine coverage trends and examined vaccination status among those hospitalized. Methods: Using the Veradigm linked claims and electronic health record dataset, we conducted a non-interventional, retrospective cohort study; three monthly cohorts included individuals aged 65+, high-risk (HR) adults (defined as adults 18+ with HR conditions and/or aged 65+), and adults aged 50–64 years who were enrolled with both medical and pharmacy coverage. We estimated monthly cumulative incidence of COVID-19 and influenza-related hospitalizations, vaccination coverage rates, and the proportion of hospitalized individuals who had received yearly updated vaccines. Results: COVID-19 hospitalizations consistently exceeded those of influenza across months and populations. Among adults aged 65+, COVID-19 hospitalization rates were 2–3 times higher than influenza in winter and 20–30 times higher during off-season months, with similar trends observed in high risk adults. COVID-19 incidence surged in summer, while influenza remained seasonally confined. Vaccination coverage for influenza peaked near 50% annually; COVID-19 coverage was lower, peaking at ~26% by December each year. Most hospitalizations occurred among unvaccinated individuals, particularly for COVID-19. Conclusions: COVID-19 continues to impose a substantial, year-round burden, particularly in older and high-risk adults, exceeding that of influenza. The high proportion of unvaccinated hospitalizations highlight a critical gap in prevention efforts and underscore the need for improved public health messaging and vaccine adoption.

## 1. Introduction

Respiratory viruses such as influenza and SARS-CoV-2 (the virus which causes COVID-19) continue to drive significant morbidity and mortality worldwide. Historically, influenza has been a perennial concern, causing an estimated 3–5 million cases of severe illness and 290,000–650,000 respiratory deaths annually, often clustering during the winter months in temperate climates, where indoor activities result in higher transmission [1]. Influenza severity fluctuates from year to year with variable morbidity and mortality, but remains fairly consistent in its seasonality such that these epidemiological trends have shaped what we consider the “respiratory season”, typically defined as October to May in the US, with peak activity from December to February [1,2]. However, when SARS-CoV-2 emerged in 2019, it quickly eclipsed previous respiratory threats, disrupting normal seasonal patterns and placing unprecedented strain on healthcare systems globally [3,4,5]. Notably, SARS-CoV-2 has not remained clustered during the “respiratory season”, resulting in summer surges and persistent healthcare demand [5,6]. As such, surveillance systems which rely on influenza seasonality to define the “respiratory season” do not reflect COVID-19 transmission patterns and will not accurately reflect the true burden of disease [7,8].

In the post-pandemic period, there continues to be discussion around whether COVID-19 or influenza provides the highest burden of disease. Some epidemiological and clinical studies suggest convergence in severity between the two infections under certain conditions—such as Omicron predominance and high population immunity—while others indicate that COVID-19 still leads to higher rates of hospitalization, ICU admission, mechanical ventilation, and death. For example, a recent study in the Veterans Affairs Health System, covering the 2023–2024 winter season, found that patients hospitalized with COVID-19 had a 35% higher adjusted 30-day mortality compared to those with influenza (5.7% vs. 4.2%) [9]. Similarly, an eight-hospital cohort in Michigan reported that COVID-19 was associated with a 30% greater risk of a composite endpoint of ICU admission, mechanical ventilation, or in-hospital death compared to influenza between 1 January 2021 and 20 July 2024 [10]. However, some surveillance network analyses have observed narrowing gaps between severe COVID-19 and influenza outcomes since mid-2022, possibly due to improved treatment, vaccination, and natural immunity [11,12].

Beyond potential differences in clinical severity, vaccine availability and uptake also present additional challenges in comparing disease burden between respiratory infectious diseases. Each year, the majority of seasonal influenza vaccines are FDA approved, and become available in the US in August. Since the advent of yearly updates, COVID-19 vaccines have had a less predictable timeline, specifically with adult vaccines being available in August–October 2022 (2022–2023 season), September–November 2023 (2023–2024 season) and August 2024 (2024–2025 season). This fluctuation leads to confusion, missed opportunities, and subsequent lower vaccine uptake [13]. It is worth noting that in early 2024 the FDA released a proposed timeline for yearly COVID-19 vaccines to align to the process for influenza vaccines, and this was adhered to in the 2024–2025 season, resulting in August approvals for both COVID-19 and the majority of influenza vaccines [14]. Vaccine uptake has also had its challenges in recent years. Looking at the last decade, self-reported influenza vaccination coverage among US adults 18 and older peaked in the 2020–2021 season at 50%, though it has declined to 45% for 2023–2024 [15]. In contrast, self-reported updated COVID-19 booster coverage has remained substantially lower—approximately 20%, among all adults for the 2023–2024 season, and as low as ~43% among those aged 65 and older, despite having the same ACIP recommendation as influenza at the time of our study, stating all persons 6 months and older should receive an updated vaccine. Additionally, those at highest risk were also optionally recommended to receive additional doses of COVID-19 vaccine based on their provider’s recommendation. This discrepancy in vaccination rates hints at the growing variety of concerns patients have regarding COVID-19 vaccines, including safety, effectiveness, risk perception, lack of trust in the government and others, and highlights the need for targeted campaigns focused on increasing rates [16].

Emerging vaccine technologies may help simplify immunization programs, reduce system inefficiency and mitigate missed vaccination opportunities. For instance, several influenza/COVID-19 combination vaccines are under development [17], including an investigational multicomponent influenza/COVID-19 mRNA vaccine which recently met noninferiority criteria when compared with co-administered standard vaccines in adults ≥50 years in a large phase 3 trial; as a result, it was granted regulatory authorization by the European Commission in April 2026 [18]. Such innovations have the potential to result in increased COVID-19 vaccination rates.

Despite these advances, uncertainties remain and diverging hypotheses persist: are COVID-19 and influenza now clinically comparable in severity due to evolving immunity, preventative strategies and treatment, or does COVID-19 still pose a disproportionate threat? Specifically, it is still unclear what impact seasonal behavioral factors, viral evolution, and waning immunity may have on hospitalization patterns.

To address these questions, our study quantified and compared monthly COVID-19 and influenza hospitalization incidence among adults aged 50–64 years, aged ≥65 years, and high-risk (HR) adults (defined as adults 18+ with HR conditions and/or aged 65+). We assessed vaccine coverage trends and examined vaccination status among those hospitalized.

## 2. Methods

We conducted a series of retrospective monthly cohort analyses to assess the incidence of hospitalization with COVID-19 or influenza burden from 1st October 2022 through 31 December 2024, in an integrated Dataset of real-world data originating from the Veradigm EHR dataset integrated with pharmacy and medical claims data, based on data availability at the time of study onset. The dataset includes data from Commercial, Medicare, and Medicaid payers; the national representativeness of the Veradigm Network EHR linked to Komodo Health claims data has been previously evaluated by Boikos et al. (2022, Vaccines) [19]. The demographic composition of the VNEHR-Komodo Integrated Dataset was compared against 2019 US Census Bureau data and the 2016 National Ambulatory Medical Care Survey (NAMCS). Geographic distribution across US Census regions and sex distributions were broadly consistent across all three data sources. Age distributions were also largely similar, though the dataset under-represents individuals under 18 years of age (10.0% vs. 22.4% in the US Census) and over-represents those over 65 years (30.0% vs. 16.0%), which is expected given that the dataset is derived from healthcare utilization sources and skews toward insured individuals who actively seek medical care. The dataset was less racially and ethnically diverse compared to the US Census, with a higher proportion of unknown/missing race data attributable to HIPAA de-identification requirements and the fact that providers are not required to record race or ethnicity in EMRs. The authors concluded that the Integrated Dataset is generally representative of the US insured population and contains the key and non-critical variables identified by the WHO as necessary for the evaluation of influenza vaccine effectiveness, supporting its use for generating real-world evidence in vaccine research.

We assessed 3 subgroups each month: all adults aged 65+, HR adults (aged 18+), and all adults aged 50–64. For each of the 27 monthly cohorts during the study period, patients were included if they met the age criteria on the first day of the indicated month in the linked dataset and were enrolled with both pharmacy and medical insurance for the indicated month. To assess HR conditions, patients were required to have evidence of at least one of the HR conditions documented at any point during the 365 days prior to the start of the indicated month. HR conditions were defined as conditions associated with increased risk of severe COVID-19 outcomes, as defined by the Centers for Disease Control and Prevention (CDC) [20]. These conditions have a high degree of overlap with those that also increase the risk of flu complications [21] and for the purpose of this study included asthma, cancer, cerebrovascular disease, chronic kidney disease, chronic liver disease, chronic lung disease, cystic fibrosis, dementia, diabetes mellitus, disability, heart conditions, HIV, mental health conditions, obesity (body mass index > 30), pregnancy, primary immunodeficiencies, respiratory tuberculosis, smoking, solid organ or stem cell transplant, and use of select immunosuppressive medications, as recorded in the database.

In each monthly cohort, we assessed the monthly cumulative incidence of hospitalizations, vaccination coverage, and vaccination rates among hospitalized patients independently for both COVID-19 and influenza. Hospitalizations were defined as a hospitalization with COVID-19 or influenza diagnosis in any position. Patients were considered up to date on their vaccination if they received a COVID-19 vaccine updated for the respiratory season after the date of regulatory approval (which changed annually: 31 August 2022; 11 September 2023; 22 August 2024) and before the next year’s approval. For influenza, all vaccinations after 15 July of the given year were assumed to be updated vaccinations as this is typically when updated influenza vaccinations are made available; specific approval dates are not widely communicated for these vaccines as they are with COVID-19 vaccines.

All results were reported descriptively. Incidence and corresponding 95% confidence intervals (CIs) were calculated per 100,000 individuals for all outcomes for each month. Incidence ratios were calculated by dividing the incidence of COVID-19 by the incidence of influenza for each month. All baseline categorical characteristics were reported as counts and percentages. No statistical testing was performed. Analyses were conducted using SAS V9.4 (SAS, Cary, NC, USA).

This study was designed, implemented, and reported in accordance with Good Pharmacoepidemiology Practice (GPP), with applicable local regulations and with the ethical principles laid down in the Declaration of Helsinki. Given that this is a retrospective database study using de-identified data, adverse event reporting was not applicable. Since this study employed a noninterventional, retrospective database using a certified Health Insurance Portability and Accountability Act-compliant de-identified research database, approval by an institutional review board was not required.

## 3. Results

A total of 47,778,112 patients were aged 18 and over, including 8,559,461 (17.9%) aged 65 or older, and 13,602,755 aged 50–64. Over 25 million patients (54.3%) of the study population had an underlying medical condition, making them high-risk for severe outcomes with COVID-19 and influenza. The most common underlying conditions were hypertension (30.9%); endocrine disorders, including Type 1 and Type 2 diabetes (25.6%); and obesity (24.2%). More than half (57.2%) of the study population was female. Demographics and clinical characteristics of the full adult population are available in Table 1. Demographic and clinical characteristics by monthly cohort are presented in Supplemental Table S1.

### 3.1. Incidence of Hospitalizations

**Figure 1:**
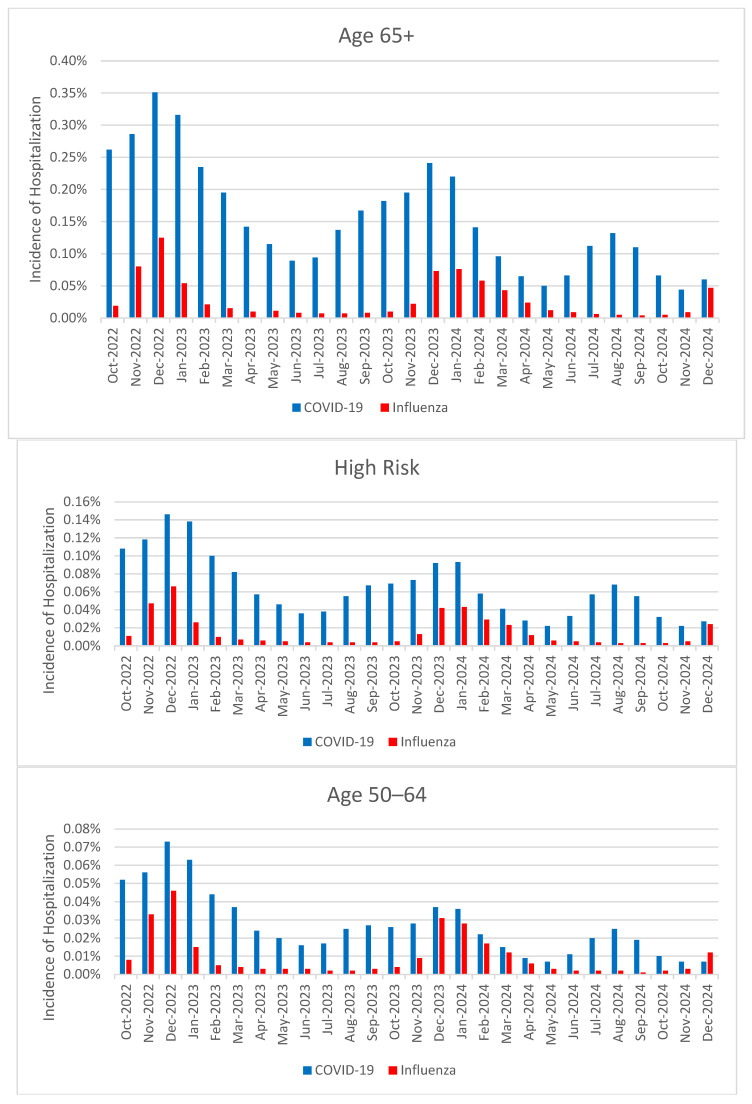
Monthly incidence of hospitalizations with COVID-19 and influenza.

In the 65+ population, the incidence of hospitalization with COVID-19 was substantially higher than the incidence of hospitalization with influenza for each of the 27 months of the study period. In the 2023 and 2024 calendar years, the lowest observed incidences of COVID-19 in June (0.089%) and May (0.050%), respectively, were similar to the highest observed incidence of hospitalization with influenza in those same years (0.073% in December 2023 and 0.076% in January 2024). Similar trends in incidence were observed in the HR population and in the 50–64 population (see Figure 1).

**Figure 2:**
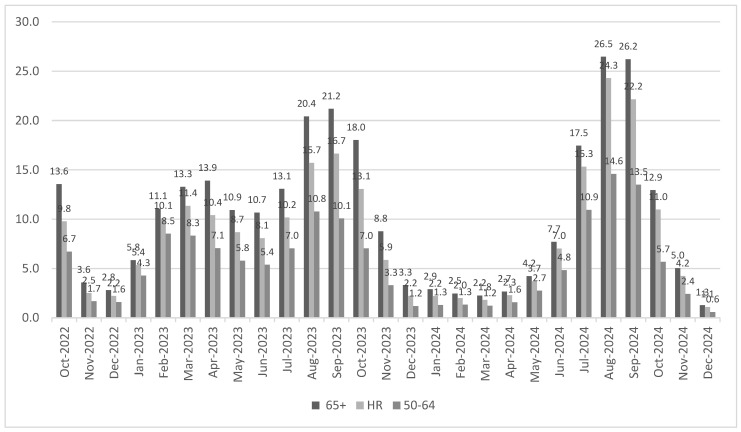
Incidence ratio of hospitalizations with COVID-19 and influenza, by age group and month.

In the population aged 65+, the incidence ratio between COVID-19 and influenza hospitalizations ranged from a low of 1.3 in the last month of the study period (December 2024) to a high of 26.5 in August 2024. Of the 27 months in the study period, more than half (*n* = 14) had an incidence ratio of at least 10× more hospitalizations with COVID-19 than influenza, and 26 out of the 27 months (96.2%) had more than 2× more hospitalizations with COVID-19 than influenza. Similar trends were seen in the HR population and in the 50–64 population (See Figure 2).

### 3.2. Vaccine Coverage Trends

Vaccine coverage rates (VCR) were consistently higher for influenza vaccination as compared with COVID-19 vaccination during the course of the study. Notably, in each season, COVID-19 vaccine availability trailed influenza vaccine availability except for the 2024–2025 season, which may have resulted in lower uptake of COVID-19 vaccines regardless of other factors. In the 65+ population, influenza VCR was similar across the three seasons, reaching 49.2% by the end of December 2022 (peaking to 54.3%), 48.9% by the end of December 2023 (peaking at 55.1%) and 45.1% by the end of December 2024. COVID-19 VCR was lower, at 24.5%, 26.3%, and 26.6% by the end of each December, rising to 34.4% and 33.8% by the summer of 2023 and 2024, respectively. In the HR population, we see the same trends. In the population aged 50–64, overall vaccination rates were lower, with 14.4% vaccinated for COVID-19 by December 2022, 11.9% by December 2023, and 12.6% by December 2025 vaccinated, and 30.2%, 26.6%, and 25.1%, respectively, at the same months for influenza. (See Table 2).

### 3.3. Vaccinations Status Among Those Hospitalized

**Figure 3:**
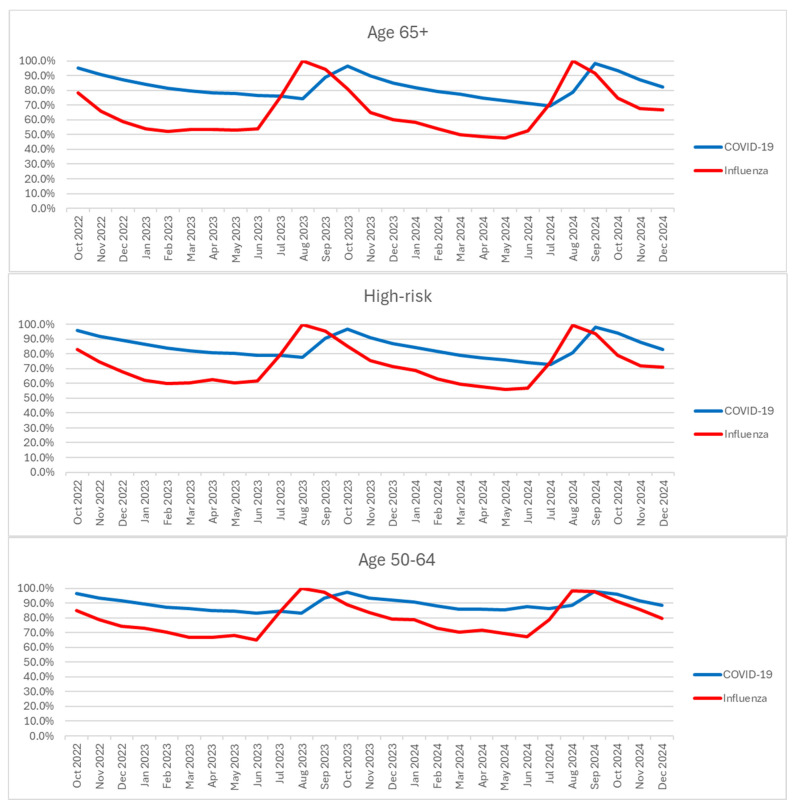
Proportion of hospitalizations who did not receive current yearly vaccination.

Among those hospitalized with COVID-19, the vast majority had not received the yearly updated COVID-19 vaccine. This was also observed for those hospitalized with influenza, but to a lesser extent. With the earlier roll-out of the influenza vaccine in the years of the study period, we also see the earlier vaccinations reflected in the data. By December 2024, approximately 80–90% of hospitalizations with COVID-19 were not up to date on their vaccinations across the age groups assessed. See Figure 3.

## 4. Discussion

In this large, retrospective analysis of over 47 million adults in the United States, we found that COVID-19 hospitalizations consistently exceeded those of influenza across all age and risk groups between October 2022 and December 2024. Among adults ≥ 65 years, COVID-19 hospitalization incidence was 2–3 times higher than influenza during winter respiratory seasons and up to 20–30 times higher during the summer months. Similar patterns were observed in adults aged 50–64, though at lower absolute rates. These findings suggest a persistent, year-round COVID-19 burden that frequently surpasses seasonal influenza, particularly in older adults. Overall since 2019, hospitalizations for COVID-19 continue to decrease, while hospitalizations for influenza can vary year to year but are not currently going through a decrease similar to COVID-19.

This study aligns with recent national and international reports showing that COVID-19 continues to impose a disproportionately greater clinical burden compared to influenza. A Veterans Affairs study reported 35% higher adjusted mortality for COVID-19 than influenza hospitalizations during the 2023–2024 winter season [9]. Likewise, CDC RESP-NET surveillance has documented age-adjusted COVID-19 hospitalization rates at nearly double those of influenza in adults [6]. European data similarly indicate that SARS-CoV-2 activity persists across longer seasonal windows than influenza, often with bimodal peaks [5]. Taken together, this reinforces that COVID-19 is not fully seasonalized and remains a major driver of hospital demand outside of traditional influenza months.

A key secondary finding of our study was the persistent gap in vaccine coverage between influenza and COVID-19. Yearly influenza vaccine uptake approached 50% among adults ≥ 65 years each winter, while COVID-19 coverage plateaued at ~25–27% by December. This discrepancy was even greater among adults aged 50–64. Importantly, most hospitalizations for both COVID-19 and influenza occurred among individuals who had not received a yearly updated vaccine, though the gap was more pronounced for COVID-19. These results highlight missed opportunities for prevention and echo CDC survey reports of low updated vaccine uptake (15–40% by age group) among US adults in 2023–2024 [22]. While these results reflect the lower vaccine update for COVID-19 as compared with influenza, it is also important to note that we cannot separate the effects of low COVID-19 vaccine uptake versus potential low influenza vaccine effectiveness in contributing to the observation. Further research to model the burden of COVID-19 and influenza under similar vaccine uptake scenarios to further elucidate the cause would be warranted.

The lower uptake of COVID-19 vaccines in the US relative to influenza likely reflects several factors, including vaccine misinformation, evolving perceptions of COVID-19 severity, and barriers to access [16,23,24]. Public health strategies emphasizing the comparable—or greater—burden of COVID-19 relative to influenza may help address these challenges. Innovations such as combined influenza/COVID-19 vaccines may also improve convenience and acceptance, thereby narrowing coverage gaps, but may also face an uphill battle due to public bias against COVID-19 vaccines [25,26].

### Strengths and Limitations

This study has several strengths, including the use of a large, linked claims–EHR dataset spanning multiple seasons, and the consistent methodology applied to both COVID-19 and influenza outcomes. However, limitations should be noted. First, the dataset used in this analysis is limited to insured individuals, and includes mainly commercial insurance. Uninsured individuals and those with certain types of insurance, such as those who receive their healthcare through the Veterans Health Administration, or who received a vaccination not communicated to the EHR or captured by an insurance claim, are not captured in this dataset. Nevertheless, this integrated dataset has been shown to be generally representative of the US and has also been used in our previous analysis to evaluate the COVID-19 burden; this analysis was highly aligned with CDC data, highlighting the volume and robustness of the dataset [19,27,28]. Second, we used a diagnosis of influenza or COVID-19 at any diagnostic position, which captures patients who are hospitalized for any reason and then test positive for influenza or COVID-19. This could inflate the incidence of both outcomes if they are heavily tested, especially among patients with non-severe disease who would not otherwise have been hospitalized. If patients are tested at different frequencies for influenza and COVID-19, the incidence of the under-tested condition will be lower due to the testing practices. Finally, the cumulative incidence was estimated each month independently, so a single episode of disease (either COVID-19 or influenza) could show up in the data as an event in subsequent months if the patient is re-admitted. This methodology allows for interpretation of the burden on the healthcare system by month, but may over-estimate total incidence of disease if months are pooled together.

## 5. Conclusions

Our findings provide timely, real-world evidence of the sustained burden of COVID-19 relative to influenza and the persistent gaps in yearly vaccination coverage. These data support continued public health messaging on the importance of updated yearly COVID-19 vaccination, particularly among older and high-risk adults, in an era where apathy has grown and the public underestimates the risk, particularly versus that of influenza. Furthermore, we continue to see COVID-19 hospitalizations outside of the traditional respiratory season and year-to-year variation in burden due to the pathogenicity of circulating strains. For these reasons, vaccination remains the best way to be prepared for unpredictable COVID-19 epidemiology.

## Figures and Tables

**Table 1:** Demographic and clinical characteristics.

	All Patients ^1^ *N* = 47,778,112	
**Female (** ***N*** **, %)**	27,344,682	57.2%
**Age, in years, at start of month (Mean, SD)**	47.5	17.6
18–49 years old (*N*, %)	25,615,896	53.6%
50–64 years old (*N*, %)	13,602,755	28.5%
65+ years old (*N*, %)	8,559,461	17.9%
**High-risk conditions during 12 months prior to the first day of the month (** ***N*** **, %)**		
**Any**	25,953,000	54.3%
Asthma	3,579,446	7.5%
Cancer	2,129,443	4.5%
Cerebrovascular disease	1,663,027	3.5%
Chronic kidney disease	2,394,086	5.0%
Chronic lung disease	2,558,477	5.4%
Chronic liver disease	418,744	0.9%
Diabetes type 1 or 2	6,751,500	14.1%
Heart conditions	4,015,532	8.4%
HIV	189,540	0.4%
Hypertension	14,740,708	30.9%
Mental health disorders	7,006,016	14.7%
Musculoskeletal Conditions	8,108,114	17.0%
Neurologic and neurodevelopment conditions	1,461,079	3.1%
Dementia	725,131	1.5%
Obesity (BMI > 30 kg/m^2^)	11,543,480	24.2%
Pregnancy	1,164,440	2.4%
Primary immunodeficiencies	366,211	0.8%
Smoking, current and former	6,441,303	13.5%
Solid organ or hematopoietic stem cell transplant	115,746	0.2%
Stroke	993,238	2.1%
Use of immunosuppressants	1,664,269	3.5%
**Other comorbid conditions during 12 months prior to the first day of the month (** ***N*** **, %)**		
Blood disorders	6,599,115	13.8%
Cystic fibrosis	9164	0.0%
Down’s syndrome	20,017	0.0%
Endocrine disorders	12,242,571	25.6%
Disabilities	3,145,460	6.6%
Metabolic disorders	16,625,637	34.8%
Morbid obesity/class III obesity (BMI > 40 kg/m^2^)	4,064,420	8.5%
Tuberculosis	14,916	0.0%

^1^ Based on first month of inclusion in the analyses.

**Table 2:** Cumulative vaccination coverage, by month and age Group.

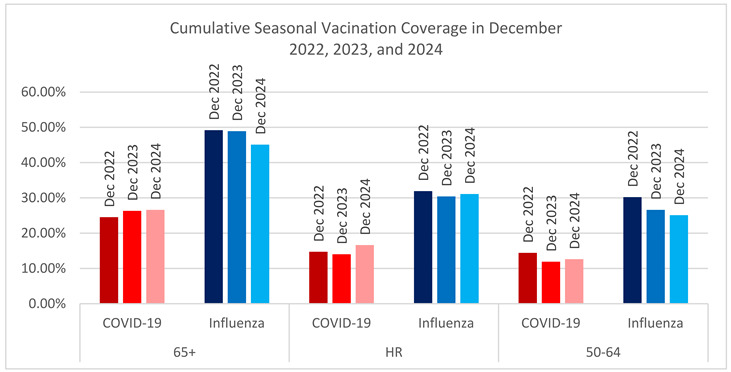						
	65+		HR		50–64	
	COVID-19	Influenza	COVID-19	Influenza	COVID-19	Influenza
October 2022	7.8%	16.2%	4.5%	9.4%	4.1%	8.0%
November 2022	18.9%	40.0%	10.9%	24.5%	10.5%	22.5%
December 2022	24.5%	49.2%	14.7%	31.9%	14.4%	30.2%
January 2023	27.9%	52.5%	17.1%	35.3%	16.2%	32.4%
February 2023	29.5%	53.6%	18.2%	36.4%	17.3%	33.5%
March 2023	30.1%	54.1%	18.6%	36.8%	17.7%	33.9%
April 2023	30.5%	54.3%	18.9%	37.0%	18.0%	34.0%
May 2023	30.8%	54.4%	19.1%	37.1%	18.2%	34.1%
June 2023	31.2%	54.4%	19.3%	37.0%	18.4%	34.1%
July 2023	31.4%	54.3%	19.4%	36.9%	18.5%	34.1%
August 2023	32.2%	0.1%	19.6%	0.0%	18.6%	0.0%
September 2023	34.4%	1.9%	21.1%	1.1%	19.5%	0.8%
October 2023	5.3%	17.5%	2.8%	10.1%	2.4%	8.1%
November 2023	19.9%	40.0%	10.4%	24.0%	8.7%	20.4%
December 2023	26.3%	48.9%	14.0%	30.4%	11.9%	26.6%
January 2024	28.6%	50.9%	16.0%	33.1%	12.8%	27.9%
February 2024	30.4%	52.5%	17.1%	34.5%	13.8%	29.3%
March 2024	31.5%	53.6%	18.4%	36.2%	14.4%	30.0%
April 2024	32.0%	53.8%	18.7%	36.4%	14.6%	30.1%
May 2024	32.4%	54.0%	19.1%	36.7%	14.7%	30.1%
June 2024	33.4%	55.0%	21.2%	39.3%	15.8%	31.5%
July 2024	33.7%	55.1%	21.4%	39.4%	15.9%	31.4%
August 2024	33.8%	0.1%	21.5%	0.1%	16.0%	0.1%
September 2024	1.0%	2.4%	0.6%	1.6%	0.5%	1.1%
October 2024	12.0%	17.7%	7.1%	11.5%	4.9%	8.6%
November 2024	22.6%	36.7%	14.2%	25.3%	10.0%	19.4%
December 2024	26.6%	45.1%	16.6%	31.1%	12.6%	25.1%

## Data Availability

The data that support the findings of this study were used under license from Veradigm and Komodo Health. Due to data use agreements and their proprietary nature, restrictions apply regarding the availability of the data. Further information is available from the corresponding author.

## Supplementary Materials

The following supporting information can be downloaded at https://www.mdpi.com/article/10.3390/vaccines14050424/s1, Table S1, Part 1–Part 3: Demographic and clinical characteristics by monthly cohort.
