# Supplementary material for: Incidence of COVID-19 and Influenza-Related Outcomes and Vaccinations in the United States, October 2022 Through December 2024
Source: Vaccines (Basel). 2026 May 8;14(5):424. doi: 10.3390/vaccines14050424 (PMC13211418; doi:10.3390/vaccines14050424)
Supplement: Supplementary file 1 [file vaccines-14-00424-s001.zip › vaccines-4254709-supplementary.pdf]

Supplemental Materials

Supplemental Table S1, Part 1. Demographic and clinical characteristics by monthly cohort.

	October 2022		November 2022		December 2022		January 2023		February 2023		March 2023		April 2023		May 2023		June 2023	
	N =	39,633,100	N =	39,505,677	N =	39,286,589	N =	38,329,001	N =	38,073,756	N =	37,814,356	N =	37,479,443	N =	37,261,649	N =	36,909,148
**Female (N, %)**	22,821,666	57.6%	22,735,976	57.6%	22,606,086	57.5%	21,978,554	57.3%	21,823,936	57.3%	21,670,006	57.3%	21,468,479	57.3%	21,334,502	57.3%	21,128,597	57.2%
**Age, in years, at start of month (Mean, SD)**	47.8	17.7	47.8	17.6	47.7	17.6	47.7	17.6	48.2	17.8	48.2	17.8	48.2	17.8	48.2	17.7	48.3	17.7
18-49 years old (N,%)	20,897,235	52.7%	20,837,047	52.7%	20,729,726	52.8%	19,892,139	51.9%	19,806,612	52.0%	19,694,592	52.1%	19,502,869	52.0%	19,386,900	52.0%	19,184,029	52.0%
50-64 years old (N,%)	11,576,691	29.2%	11,564,474	29.3%	11,518,320	29.3%	10,983,602	28.7%	10,934,936	28.7%	10,896,467	28.8%	10,825,660	28.9%	10,783,806	28.9%	10,716,827	29.0%
65+ years old (N,%)*	7,159,174	18.1%	7,104,156	18.0%	7,038,543	17.9%	7,453,260	19.4%	7,332,208	19.3%	7,223,297	19.1%	7,150,914	19.1%	7,090,943	19.0%	7,008,292	19.0%
**High-risk conditions during 12 months prior to the first day of the month (N, %)**																		
**Any**	25,902,932	65.4%	25,779,612	65.3%	24,950,943	63.5%	24,848,034	64.8%	24,745,195	65.0%	24,579,037	65.0%	24,492,280	65.3%	24,342,370	65.3%	23,817,893	64.5%
Asthma	3,180,865	8.0%	3,181,029	8.1%	3,174,178	8.1%	3,082,461	8.0%	3,070,134	8.1%	3,062,814	8.1%	3,041,334	8.1%	3,033,405	8.1%	3,016,948	8.2%
Cancer	1,868,436	4.7%	1,870,697	4.7%	1,863,703	4.7%	1,791,234	4.7%	1,787,127	4.7%	1,782,388	4.7%	1,776,804	4.7%	1,778,875	4.8%	1,778,245	4.8%
Cerebrovascular disease	1,461,145	3.7%	1,462,878	3.7%	1,457,275	3.7%	1,381,560	3.6%	1,380,420	3.6%	1,378,152	3.6%	1,373,759	3.7%	1,374,854	3.7%	1,376,986	3.7%
Chronic kidney disease	2,103,981	5.3%	2,110,956	5.3%	2,107,508	5.4%	1,995,182	5.2%	1,988,957	5.2%	1,983,501	5.2%	1,983,424	5.3%	1,990,492	5.3%	1,995,686	5.4%
Chronic lung disease	2,253,720	5.7%	2,250,043	5.7%	2,240,394	5.7%	2,103,312	5.5%	2,086,308	5.5%	2,073,149	5.5%	2,061,508	5.5%	2,058,086	5.5%	2,052,560	5.6%

Chronic liver disease	368,062	0.9%	368,562	0.9%	367,320	0.9%	348,573	0.9%	349,576	0.9%	349,170	0.9%	347,784	0.9%	348,367	0.9%	348,789	0.9%
Diabetes type 1 or 2	5,927,603	15.0%	5,925,919	15.0%	5,903,919	15.0%	5,618,023	14.7%	5,606,845	14.7%	5,589,926	14.8%	5,567,242	14.9%	5,565,416	14.9%	5,556,388	15.1%
Heart conditions	3,522,201	8.9%	3,525,782	8.9%	3,516,227	9.0%	3,340,726	8.7%	3,333,396	8.8%	3,328,797	8.8%	3,324,622	8.9%	3,330,936	8.9%	3,335,903	9.0%
HIV	165,176	0.4%	164,563	0.4%	163,619	0.4%	155,499	0.4%	154,832	0.4%	154,917	0.4%	153,067	0.4%	152,163	0.4%	151,352	0.4%
Hypertension	13,003,183	32.8%	12,991,466	32.9%	12,938,851	32.9%	12,332,866	32.2%	12,306,556	32.3%	12,274,622	32.5%	12,229,363	32.6%	12,215,318	32.8%	12,178,788	33.0%
Mental health disorders	6,172,895	15.6%	6,137,521	15.5%	6,084,738	15.5%	5,818,823	15.2%	5,794,055	15.2%	5,759,684	15.2%	5,699,700	15.2%	5,669,775	15.2%	5,633,784	15.3%
Musculoskeletal Conditions	7,188,395	18.1%	7,192,715	18.2%	7,159,893	18.2%	6,817,400	17.8%	6,813,365	17.9%	6,792,653	18.0%	6,758,128	18.0%	6,753,636	18.1%	6,749,715	18.3%
Neurologic and neurodevelopment conditions	1,284,004	3.2%	1,281,265	3.2%	1,272,934	3.2%	1,219,462	3.2%	1,211,528	3.2%	1,202,850	3.2%	1,194,271	3.2%	1,190,445	3.2%	1,186,672	3.2%
Dementia	634,421	1.6%	632,650	1.6%	628,203	1.6%	583,881	1.5%	578,113	1.5%	573,288	1.5%	570,436	1.5%	568,439	1.5%	567,154	1.5%
Obesity (BMI >30 kg/m^2)	10,149,200	25.6%	10,146,193	25.7%	10,097,937	25.7%	9,679,200	25.3%	9,675,834	25.4%	9,660,081	25.5%	9,606,728	25.6%	9,592,575	25.7%	9,565,801	25.9%
Pregnancy	1,028,545	2.6%	1,012,639	2.6%	997,788	2.5%	939,978	2.5%	934,697	2.5%	924,785	2.4%	909,327	2.4%	898,827	2.4%	885,045	2.4%
Primary immunodeficiencies	321,549	0.8%	330,280	0.8%	336,504	0.9%	327,621	0.9%	329,195	0.9%	333,588	0.9%	337,832	0.9%	343,999	0.9%	350,155	0.9%
Smoking, current and former	5,734,679	14.5%	5,716,608	14.5%	5,681,265	14.5%	5,365,617	14.0%	5,341,299	14.0%	5,320,560	14.1%	5,274,488	14.1%	5,249,560	14.1%	5,216,686	14.1%
Solid organ or hematopoietic stem cell transplant	97,204	0.2%	97,424	0.2%	97,211	0.2%	93,490	0.2%	93,054	0.2%	92,666	0.2%	92,278	0.2%	92,396	0.2%	92,253	0.2%
Stroke	865,896	2.2%	866,545	2.2%	862,587	2.2%	813,980	2.1%	813,245	2.1%	810,231	2.1%	805,658	2.1%	804,899	2.2%	805,122	2.2%
Use of immunosuppressants	1,455,731	3.7%	1,462,642	3.7%	1,461,483	3.7%	1,411,049	3.7%	1,413,032	3.7%	1,414,066	3.7%	1,412,339	3.8%	1,415,684	3.8%	1,418,081	3.8%
**Other comorbid conditions during 12 months prior to**																		

**the first day of the month (N, %)**																		
Blood disorders	5,863,595	14.8%	5,868,067	14.9%	5,848,221	14.9%	5,577,441	14.6%	5,584,762	14.7%	5,578,666	14.8%	5,548,781	14.8%	5,554,922	14.9%	5,561,362	15.1%
Cystic fibrosis	8,097	0.0%	8,049	0.0%	8,019	0.0%	7,741	0.0%	7,678	0.0%	7,584	0.0%	7,497	0.0%	7,456	0.0%	7,397	0.0%
Down's syndrome	18,111	0.0%	17,938	0.0%	17,702	0.0%	17,378	0.0%	17,213	0.0%	17,024	0.0%	16,717	0.0%	16,612	0.0%	16,634	0.0%
Endocrine disorders	10,794,603	27.2%	10,796,036	27.3%	10,757,223	27.4%	10,287,778	26.8%	10,279,614	27.0%	10,258,805	27.1%	10,214,977	27.3%	10,213,306	27.4%	10,199,601	27.6%
Disabilities	2,766,906	7.0%	2,774,008	7.0%	2,764,696	7.0%	2,723,881	7.1%	2,734,472	7.2%	2,733,120	7.2%	2,715,721	7.2%	2,711,899	7.3%	2,705,048	7.3%
Metabolic disorders	14,787,359	37.3%	14,802,562	37.5%	14,762,854	37.6%	14,111,546	36.8%	14,120,316	37.1%	14,112,312	37.3%	14,070,354	37.5%	14,085,597	37.8%	14,084,480	38.2%
Morbid obesity/class III obesity (BMI >40 kg/m^2)	3,586,996	9.1%	3,593,215	9.1%	3,581,903	9.1%	3,425,590	8.9%	3,426,684	9.0%	3,424,779	9.1%	3,412,129	9.1%	3,414,132	9.2%	3,413,679	9.2%
Tuberculosis	13,340	0.0%	13,264	0.0%	13,119	0.0%	12,388	0.0%	12,322	0.0%	12,257	0.0%	12,009	0.0%	11,969	0.0%	12,063	0.0%

Supplemental Table S1, Part 2. Demographic and clinical characteristics by monthly cohort

	July 2023		August 2023		September 2023		October 2023		November 2023		December 2023		January 2024		February 2024		March 2024	
	N =	36,140,582	N =	34,969,944	N =	27,419,930	N =	27,293,396	N =	26,995,848	N =	26,775,121	N =	25,698,512	N =	25,340,274	N =	21,549,587
**Female (N, %)**	20,679,922	57.2%	19,969,752	57.1%	15,473,201	56.4%	15,402,702	56.4%	15,229,360	56.4%	15,103,077	56.4%	14,462,348	56.3%	14,255,538	56.3%	12,165,168	56.5%
**Age, in years, at start of month (Mean, SD)**	48.2	17.7	48.1	17.6	48.5	17.6	48.5	17.6	48.5	17.5	48.5	17.5	49.1	17.8	49.1	17.7	50.0	18.0
18-49 years old (N,%)	18,818,277	52.1%	18,281,083	52.3%	14,078,783	51.3%	14,015,971	51.4%	13,897,316	51.5%	13,793,895	51.5%	12,918,381	50.3%	12,766,828	50.4%	10,476,010	48.6%
50-64 years old (N,%)	10,574,907	29.3%	10,256,589	29.3%	8,175,057	29.8%	8,150,739	29.9%	8,081,479	29.9%	8,032,183	30.0%	7,507,741	29.2%	7,418,243	29.3%	6,192,945	28.7%
65+ years old (N,%)*	6,747,398	18.7%	6,432,272	18.4%	5,166,090	18.8%	5,126,686	18.8%	5,017,053	18.6%	4,949,043	18.5%	5,272,390	20.5%	5,155,203	20.3%	4,880,632	22.6%
**High-risk conditions during**																		

**12 months prior to the first day of the month (N, %)**																		
**Any**	22,995,768	63.6%	18,060,471	51.6%	17,977,242	65.6%	17,818,243	65.3%	17,693,882	65.5%	16,873,086	63.0%	16,660,854	64.8%	14,432,913	57.0%	14,275,982	66.2%
Asthma	2,946,534	8.2%	2,816,034	8.1%	2,151,638	7.8%	2,136,386	7.8%	2,121,361	7.9%	2,106,567	7.9%	2,001,411	7.8%	1,977,848	7.8%	1,692,224	7.9%
Cancer	1,739,283	4.8%	1,678,341	4.8%	1,381,081	5.0%	1,376,807	5.0%	1,365,373	5.1%	1,360,309	5.1%	1,294,253	5.0%	1,278,740	5.0%	1,159,497	5.4%
Cerebrovascular disease	1,342,237	3.7%	1,285,222	3.7%	986,206	3.6%	982,777	3.6%	975,772	3.6%	972,125	3.6%	920,804	3.6%	908,274	3.6%	828,745	3.8%
Chronic kidney disease	1,949,012	5.4%	1,867,405	5.3%	1,422,156	5.2%	1,422,997	5.2%	1,414,410	5.2%	1,412,746	5.3%	1,338,541	5.2%	1,321,324	5.2%	1,219,568	5.7%
Chronic lung disease	1,996,232	5.5%	1,916,503	5.5%	1,395,161	5.1%	1,390,195	5.1%	1,376,936	5.1%	1,369,451	5.1%	1,297,406	5.0%	1,274,011	5.0%	1,138,703	5.3%
Chronic liver disease	341,670	0.9%	330,024	0.9%	242,477	0.9%	241,443	0.9%	240,134	0.9%	239,481	0.9%	225,017	0.9%	221,848	0.9%	190,321	0.9%
Diabetes type 1 or 2	5,446,152	15.1%	5,198,381	14.9%	3,887,959	14.2%	3,877,209	14.2%	3,849,266	14.3%	3,834,149	14.3%	3,620,978	14.1%	3,583,468	14.1%	3,131,166	14.5%
Heart conditions	3,259,747	9.0%	3,138,998	9.0%	2,442,289	8.9%	2,438,606	8.9%	2,418,389	9.0%	2,410,278	9.0%	2,290,054	8.9%	2,260,318	8.9%	2,051,116	9.5%
HIV	148,361	0.4%	140,278	0.4%	95,158	0.3%	94,405	0.3%	93,305	0.3%	92,377	0.3%	86,906	0.3%	85,060	0.3%	71,006	0.3%
Hypertension	11,924,070	33.0%	11,493,157	32.9%	8,986,032	32.8%	8,952,844	32.8%	8,882,362	32.9%	8,839,956	33.0%	8,342,870	32.5%	8,248,231	32.5%	7,257,282	33.7%
Mental health disorders	5,487,669	15.2%	5,281,329	15.1%	3,965,811	14.5%	3,937,700	14.4%	3,913,194	14.5%	3,883,105	14.5%	3,661,238	14.2%	3,600,609	14.2%	3,049,584	14.2%
Musculoskeletal Conditions	6,604,283	18.3%	6,364,696	18.2%	5,036,253	18.4%	5,014,357	18.4%	4,978,938	18.4%	4,951,098	18.5%	4,668,977	18.2%	4,613,795	18.2%	4,112,998	19.1%
Neurologic and neurodevelopment conditions	1,149,255	3.2%	1,097,719	3.1%	807,432	2.9%	802,340	2.9%	792,747	2.9%	786,853	2.9%	751,115	2.9%	736,748	2.9%	659,817	3.1%
Dementia	544,884	1.5%	511,878	1.5%	369,685	1.3%	366,117	1.3%	361,672	1.3%	359,849	1.3%	339,030	1.3%	332,457	1.3%	312,275	1.4%
Obesity (BMI >30 kg/m^2)	9,390,828	26.0%	9,100,114	26.0%	7,065,015	25.8%	7,026,935	25.7%	6,992,732	25.9%	6,951,069	26.0%	6,550,234	25.5%	6,475,721	25.6%	5,554,042	25.8%
Pregnancy	860,047	2.4%	819,360	2.3%	593,252	2.2%	583,826	2.1%	577,577	2.1%	569,574	2.1%	525,905	2.0%	519,428	2.0%	421,228	2.0%
Primary immunodeficienci	346,598	1.0%	341,470	1.0%	261,174	1.0%	263,277	1.0%	265,142	1.0%	268,455	1.0%	259,246	1.0%	256,637	1.0%	233,040	1.1%

es																		
Smoking, current and former	5,078,900	14.1%	4,900,524	14.0%	3,595,987	13.1%	3,564,284	13.1%	3,531,767	13.1%	3,503,372	13.1%	3,296,968	12.8%	3,240,383	12.8%	2,748,405	12.8%
Solid organ or hematopoietic stem cell transplant	90,487	0.3%	87,018	0.2%	67,138	0.2%	66,953	0.2%	66,170	0.2%	65,922	0.2%	62,970	0.2%	62,127	0.2%	54,214	0.3%
Stroke	783,654	2.2%	751,787	2.1%	558,622	2.0%	555,872	2.0%	550,806	2.0%	547,764	2.0%	517,760	2.0%	509,051	2.0%	458,413	2.1%
Use of immunosuppressants	1,396,260	3.9%	1,358,702	3.9%	1,110,705	4.1%	1,109,567	4.1%	1,107,275	4.1%	1,104,428	4.1%	1,047,849	4.1%	1,044,228	4.1%	924,427	4.3%
**Other comorbid conditions during 12 months prior to the first day of the month (N, %)**																		
Blood disorders	5,454,196	15.1%	5,232,844	15.0%	3,978,081	14.5%	3,963,169	14.5%	3,948,285	14.6%	3,934,790	14.7%	3,716,402	14.5%	3,666,726	14.5%	3,222,321	15.0%
Cystic fibrosis	7,185	0.0%	6,924	0.0%	5,206	0.0%	5,139	0.0%	5,103	0.0%	5,041	0.0%	4,802	0.0%	4,727	0.0%	3,954	0.0%
Down's syndrome	16,118	0.0%	15,644	0.0%	9,318	0.0%	9,249	0.0%	9,130	0.0%	9,030	0.0%	8,856	0.0%	8,700	0.0%	6,986	0.0%
Endocrine disorders	10,005,852	27.7%	9,629,895	27.5%	7,518,602	27.4%	7,493,326	27.5%	7,446,573	27.6%	7,415,092	27.7%	6,999,020	27.2%	6,925,036	27.3%	6,027,842	28.0%
Disabilities	2,650,898	7.3%	2,574,440	7.4%	2,044,129	7.5%	2,030,614	7.4%	2,025,141	7.5%	2,016,338	7.5%	1,935,386	7.5%	1,920,012	7.6%	1,624,500	7.5%
Metabolic disorders	13,830,288	38.3%	13,368,918	38.2%	10,522,066	38.4%	10,492,381	38.4%	10,433,993	38.7%	10,395,599	38.8%	9,795,430	38.1%	9,689,824	38.2%	8,532,348	39.6%
Morbid obesity/class III obesity (BMI >40 kg/m^2)	3,357,890	9.3%	3,266,278	9.3%	2,509,197	9.2%	2,497,258	9.1%	2,488,162	9.2%	2,476,747	9.3%	2,329,422	9.1%	2,298,487	9.1%	1,959,837	9.1%
Tuberculosis	11,747	0.0%	11,172	0.0%	7,196	0.0%	7,080	0.0%	7,082	0.0%	7,043	0.0%	6,511	0.0%	6,457	0.0%	5,636	0.0%

Supplemental Table S1, Part 3. Demographic and clinical characteristics by monthly cohort.

	April 2024		May 2024		June 2024		July 2024		August 2024		September 2024		October 2024		November 2024		December 2024	
	N =	21,327,312	N =	20,451,350	N =	15,186,466	N =	14,762,467	N =	14,143,335	N =	13,510,985	N =	12,083,613	N =	9,932,380	N =	7,950,244
**Female (N, %)**	12,034,950	56.4%	11,562,484	56.5%	8,698,748	57.3%	8,450,578	57.2%	8,118,253	57.4%	7,754,139	57.4%	6,942,799	57.5%	5,674,108	57.1%	4,513,638	56.8%
**Age, in years, at start of month (Mean, SD)**	50.0	18.1	50.2	18.1	51.9	18.8	52.0	18.8	52.1	18.8	52.1	18.8	51.7	18.7	52.5	18.7	51.4	18.2
18-49 years old (N,%)	10,365,032	48.6%	9,866,391	48.2%	6,864,189	45.2%	6,645,582	45.0%	6,330,358	44.8%	6,057,303	44.8%	5,532,331	45.8%	4,377,368	44.1%	3,673,048	46.2%
50-64 years old (N,%)	6,126,550	28.7%	5,832,308	28.5%	3,959,159	26.1%	3,838,715	26.0%	3,687,012	26.1%	3,521,334	26.1%	3,171,759	26.2%	2,620,359	26.4%	2,209,599	27.8%
65+ years old (N,%)*	4,835,730	22.7%	4,752,651	23.2%	4,363,118	28.7%	4,278,170	29.0%	4,125,965	29.2%	3,932,348	29.1%	3,379,523	28.0%	2,934,653	29.5%	2,067,597	26.0%
**High-risk conditions during 12 months prior to the first day of the month (N, %)**																		
**Any**	12,034,950	56.4%	11,562,484	56.5%	8,698,748	57.3%	8,450,578	57.2%	8,118,253	57.4%	7,754,139	57.4%	6,942,799	57.5%	5,674,108	57.1%	4,513,638	56.8%
Asthma	1,666,081	7.8%	1,607,565	7.9%	1,260,697	8.3%	1,222,542	8.3%	1,176,917	8.3%	1,122,018	8.3%	989,735	8.2%	779,843	7.9%	588,590	7.4%
Cancer	1,149,784	5.4%	1,124,909	5.5%	941,239	6.2%	923,640	6.3%	895,750	6.3%	857,526	6.3%	746,531	6.2%	638,481	6.4%	463,587	5.8%
Cerebrovascular disease	821,573	3.9%	808,532	4.0%	708,934	4.7%	695,682	4.7%	675,907	4.8%	648,372	4.8%	554,477	4.6%	461,831	4.6%	324,967	4.1%
Chronic kidney disease	1,215,412	5.7%	1,205,490	5.9%	1,080,420	7.1%	1,068,641	7.2%	1,044,584	7.4%	1,007,509	7.5%	866,179	7.2%	734,791	7.4%	523,161	6.6%
Chronic lung disease	1,126,701	5.3%	1,102,488	5.4%	961,707	6.3%	941,750	6.4%	910,360	6.4%	869,449	6.4%	735,124	6.1%	594,069	6.0%	409,399	5.1%
Chronic liver disease	188,340	0.9%	183,537	0.9%	149,040	1.0%	145,309	1.0%	141,215	1.0%	134,774	1.0%	118,433	1.0%	94,350	0.9%	68,427	0.9%
Diabetes type 1 or 2	3,106,851	14.6%	3,028,437	14.8%	2,461,353	16.2%	2,406,965	16.3%	2,339,153	16.5%	2,241,182	16.6%	1,961,507	16.2%	1,632,970	16.4%	1,242,444	15.6%
Heart conditions	2,037,590	9.6%	2,001,738	9.8%	1,716,733	11.3%	1,689,566	11.4%	1,643,367	11.6%	1,577,222	11.7%	1,358,430	11.2%	1,149,316	11.6%	821,690	10.3%

HIV	70,085	0.3%	68,485	0.3%	47,618	0.3%	46,040	0.3%	44,680	0.3%	43,411	0.3%	40,538	0.3%	29,858	0.3%	23,635	0.3%
Hypertension	7,183,502	33.7%	6,977,976	34.1%	5,591,149	36.8%	5,467,921	37.0%	5,298,117	37.5%	5,075,028	37.6%	4,445,485	36.8%	3,726,847	37.5%	2,812,111	35.4%
Mental health disorders	3,002,402	14.1%	2,890,140	14.1%	2,247,104	14.8%	2,184,633	14.8%	2,102,527	14.9%	1,996,828	14.8%	1,776,093	14.7%	1,390,106	14.0%	1,005,786	12.7%
Musculoskeletal Conditions	4,068,685	19.1%	3,967,714	19.4%	3,275,302	21.6%	3,207,926	21.7%	3,109,272	22.0%	2,972,228	22.0%	2,595,289	21.5%	2,181,778	22.0%	1,609,222	20.2%
Neurologic and neurodevelopment conditions	651,940	3.1%	635,849	3.1%	556,312	3.7%	542,234	3.7%	520,359	3.7%	492,237	3.6%	411,413	3.4%	328,475	3.3%	223,854	2.8%
Dementia	310,812	1.5%	307,955	1.5%	292,368	1.9%	288,200	2.0%	280,541	2.0%	270,054	2.0%	225,150	1.9%	186,193	1.9%	124,127	1.6%
Obesity (BMI >30 kg/m²)	5,472,009	25.7%	5,263,996	25.7%	3,949,898	26.0%	3,844,983	26.0%	3,698,553	26.2%	3,532,157	26.1%	3,107,602	25.7%	2,513,843	25.3%	1,969,842	24.8%
Pregnancy	413,333	1.9%	396,157	1.9%	297,922	2.0%	286,064	1.9%	271,069	1.9%	256,302	1.9%	235,926	2.0%	168,822	1.7%	135,615	1.7%
Primary immunodeficiencies	233,287	1.1%	231,761	1.1%	199,380	1.3%	196,997	1.3%	188,529	1.3%	184,454	1.4%	166,023	1.4%	146,094	1.5%	101,701	1.3%
Smoking, current and former	2,705,166	12.7%	2,606,439	12.7%	2,062,917	13.6%	2,001,369	13.6%	1,914,608	13.5%	1,819,271	13.5%	1,561,431	12.9%	1,173,948	11.8%	822,517	10.3%
Solid organ or hematopoietic stem cell transplant	53,742	0.3%	52,341	0.3%	43,803	0.3%	42,902	0.3%	41,494	0.3%	39,728	0.3%	35,553	0.3%	28,960	0.3%	20,351	0.3%
Stroke	453,450	2.1%	444,855	2.2%	387,632	2.6%	378,887	2.6%	367,519	2.6%	351,225	2.6%	301,878	2.5%	245,654	2.5%	172,634	2.2%
Use of immunosuppressants	919,205	4.3%	895,693	4.4%	707,931	4.7%	694,598	4.7%	676,239	4.8%	645,620	4.8%	575,105	4.8%	483,776	4.9%	364,626	4.6%
**Other comorbid conditions during 12 months prior to the first day of the month (N, %)**																				
Blood disorders	3,187,198	14.9%	3,110,404	15.2%	2,515,194	16.6%	2,460,631	16.7%	2,387,632	16.9%	2,293,902	17.0%	2,017,043	16.7%	1,667,218	16.8%	1,250,717	15.7%
Cystic fibrosis	3,861	0.0%	3,719	0.0%	2,706	0.0%	2,683	0.0%	2,592	0.0%	2,499	0.0%	2,274	0.0%	1,834	0.0%	1,392	0.0%
Down's syndrome	6,861	0.0%	6,682	0.0%	5,812	0.0%	5,638	0.0%	5,361	0.0%	5,125	0.0%	4,717	0.0%	3,182	0.0%	1,716	0.0%
Endocrine disorders	5,970,625	28.0%	5,796,640	28.3%	4,577,796	30.1%	4,474,144	30.3%	4,341,243	30.7%	4,163,929	30.8%	3,676,177	30.4%	3,093,281	31.1%	2,365,887	29.8%

Disabilities	1,602,146	7.5%	1,545,147	7.6%	1,141,933	7.5%	1,112,113	7.5%	1,074,989	7.6%	1,024,898	7.6%	923,635	7.6%	739,264	7.4%	564,293	7.1%
Metabolic disorders	8,446,619	39.6%	8,205,570	40.1%	6,476,888	42.6%	6,329,661	42.9%	6,137,131	43.4%	5,889,420	43.6%	5,184,191	42.9%	4,384,790	44.1%	3,352,904	42.2%
Morbid obesity/class III obesity (BMI >40 kg/m²)	1,929,127	9.0%	1,860,811	9.1%	1,416,002	9.3%	1,378,116	9.3%	1,327,104	9.4%	1,257,565	9.3%	1,106,628	9.2%	876,141	8.8%	670,012	8.4%
Tuberculosis	5,539	0.0%	5,453	0.0%	4,423	0.0%	4,284	0.0%	4,142	0.0%	3,955	0.0%	3,504	0.0%	2,743	0.0%	2,076	0.0%
